# The Extracellular, Cellular, and Nuclear Stiffness, a Trinity in the Cancer Resistome—A Review

**DOI:** 10.3389/fonc.2019.01376

**Published:** 2019-12-06

**Authors:** Sara Sofia Deville, Nils Cordes

**Affiliations:** ^1^OncoRay – National Center for Radiation Research in Oncology, Faculty of Medicine and University Hospital Carl Gustav Carus, Helmholtz-Zentrum Dresden - Rossendorf, Technische Universität Dresden, Dresden, Germany; ^2^Department of Radiation Oncology, University Hospital Carl Gustav Carus, Technische Universität Dresden, Dresden, Germany; ^3^Helmholtz-Zentrum Dresden - Rossendorf, Institute of Radiooncology - OncoRay, Dresden, Germany; ^4^German Cancer Consortium (DKTK), Dresden, Germany; ^5^Germany German Cancer Research Center (DKFZ), Heidelberg, Germany

**Keywords:** stiffness, extracellular matrix, cancer resistome, radio(chemo)resistance, cell-extracellular matrix interaction, focal adhesions, solid stress

## Abstract

Alterations in mechano-physiological properties of a tissue instigate cancer burdens in parallel to common genetic and epigenetic alterations. The chronological and mechanistic interrelation between the various extra- and intracellular aspects remains largely elusive. Mechano-physiologically, integrins and other cell adhesion molecules present the main mediators for transferring and distributing forces between cells and the extracellular matrix (ECM). These cues are channeled via focal adhesion proteins, termed the focal adhesomes, to cytoskeleton and nucleus and vice versa thereby affecting the pathophysiology of multicellular cancer tissues. In combination with simultaneous activation of diverse downstream signaling pathways, the phenotypes of cancer cells are created and driven characterized by deregulated transcriptional and biochemical cues that elicit the hallmarks of cancer. It, however, remains unclear how elastostatic modifications, i.e., stiffness, in the extracellular, intracellular, and nuclear compartment contribute and control the resistance of cancer cells to therapy. In this review, we discuss how stiffness of unique tumor components dictates therapy response and what is known about the underlying molecular mechanisms.

## Introduction

Stiffness refers to the rigidity of a material or the extent to which the material can resist to deformation or deflection in response to an applied force (1). Typically, stiffness depends on properties of the material such as the composition and organization of the building elements. A stiff as compared to a flexible structure is less susceptible to deform under an external load and, consequently, apt to develop greater stress.

Generally, the composition of the extracellular matrix (ECM) determines the stiffness of a tissue (2, 3). Cells are surrounded by ECM providing structural and biochemical support. Eventually, these interactions present the fundamental organization unit of multicellular complex development into tissues. The ECM comprises two classes of macromolecules: polysaccharide chains and fibrillar proteins (4). The polysaccharide chains are covalently bound to transmembrane proteins and assemble into proteoglycans. The fibrillar proteins like collagens, fibronectin, elastin, and laminins have structural functions and serve as ligands for cell adhesion molecules. The proteoglycans form a gel-like structure in which the fibrillar proteins are embedded. Mesenchymal cells such as fibroblasts are responsible for the production and secretion of ECM proteins (5–8). The ECM is constantly reorganized and its dynamic is modulated by growth factors, cytokines, hormones, and extracorporal factors influencing significantly tissue physiology, morphology, homeostasis, and repair (9). A primary source of ECM restructuring is re-synthesis or proteolytic degradation by matrix metalloproteinases (MMPs) (10, 11). Several studies indicate that the spatio-temporal organization and dynamic re-modulation of the ECM has extensive biological implications for tumorigenesis promotion, progression, and metastasis.

In general, the outgrowth of a tumor produces an additional physical pressure, also defined as stress, on the host tissue and this is reciprocally balanced by the physical stress generated by the host tissue on the tumor. To overcome the stress enforced by the host tissue, tumor stiffening is essential for allowing host tissue displacement and growth in size (12). Tumors modulate their surrounding microenvironment including ECM, which results in alterations of tissue stiffness, porosity, and organization (13). A number of studies demonstrated specific changes in the mechanical properties of tumors over the time of their progression. When measured as single component, cancer cells and their nuclei become softer compared to normal cells (14, 15) suggesting a dis-regulation of cellular signaling pathways, cell proliferation, migration, survival, and treatment resistance (16, 17).

**Figure 1:**
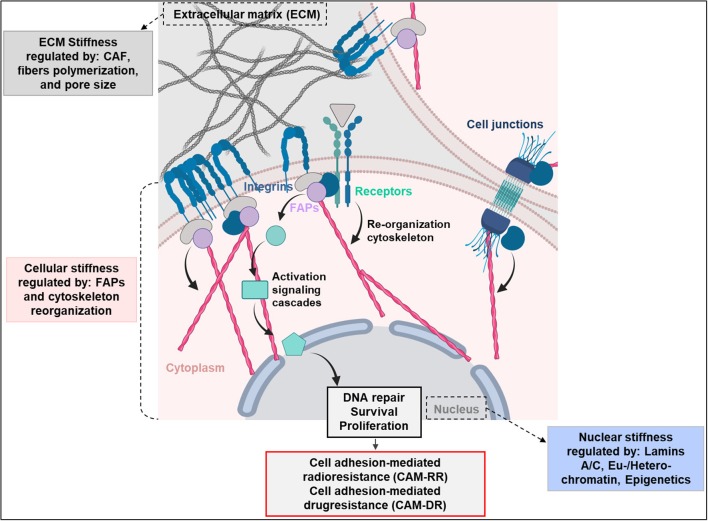
Extracellular matrix (ECM), cellular end nuclear stiffness are regulated by several factors. The ECM remodeling is highly dependent on cancer associated fibroblasts (CAFs). The cell stiffness instead is regulated by integrins and focal adhesion proteins (FAPs), which contribute to cancer radio- and drug-resistance by mediating cell adhesion to the extracellular matrix. Upon cell adhesion to ECM, integrins induce pro-survival signaling cascades mediating radiotherapy- and drug-resistance (CAM-RR and CAM-DR). Finally, the nuclear stiffness is regulated by the levels of lamin-A/C and chromatin condensation. Created with BioRender.

Fundamental for cell stiffness and mechanical forces are focal adhesions, serving as nexus between cytoskeleton and ECM (18–20). Cell adhesion elicits activation of different cytoplasmic signaling pathways for co-regulation of pro-survival mechanisms (5–8). Key mediators of this adhesion are integrins, an essential family among cell adhesion molecules. ECM reorganization drives significant changes in the integrin-mediated signaling pathways fundamental for tumor development and response to chemo- and radiotherapy (21–24). Various studies in normal (e.g., human fibroblasts and keratinocytes) and tumor cells (e.g., glioblastoma, pancreatic carcinomas, bronchial carcinomas, melanomas, breast cancers) documented adhesion to ECM to enhance resistance to ionizing radiation, chemotherapy, and molecular therapies (25, 26). These mechanisms are referred to as cell adhesion-mediated radioresistance (CAM-RR) and cell adhesion-mediated drug resistance (CAM-DR) (Figure 1) (25, 27).

This review gives insights into recent findings of how tissue, cellular, and nuclear stiffness are associated with therapy resistance and discusses the underlying mechanisms.

## Regulation of Cancer Therapy Resistance Through ECM Remodeling and Stiffness

During tumor progression, cellular and genomic alterations occur, which are accompanied by changes in mechanical properties in the intracellular- and extracellular environment. The ECM is a key component of the tumor microenvironment, which interacts with cancer cells and regulates signaling cascades through focal adhesion proteins (FAPs) (28–30).

**Figure 2:**
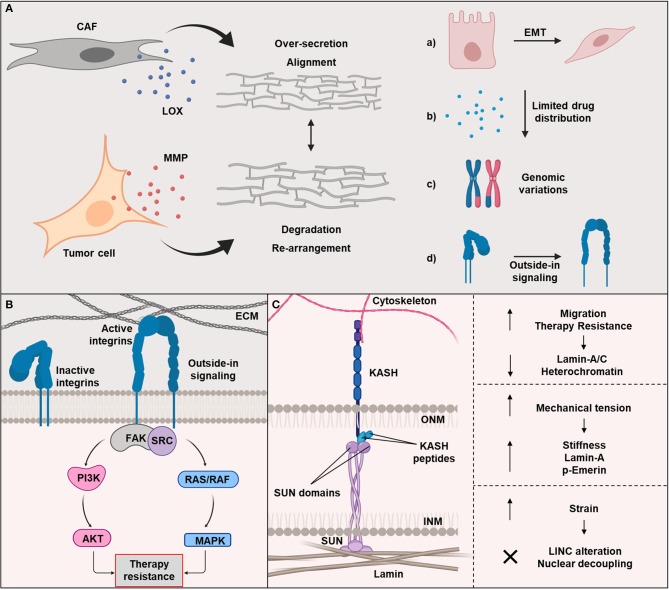
**(A)** The extracellular matrix (ECM) secretion depends mainly on cancer associated fibroblasts (CAFs). The dynamic reorganization of ECM is regulated by matrix metalloproteinase (MMP)-dependent matrix degradation and lysyl oxidase (LOX)-dependent ECM crosslinking. Changes in ECM and stiffening leads to: (a) epithelial-to-mesenchymal transition (EMT) enhancing cell migration and invasion, (b) limited drugs distribution, (c) genomic alterations resulting in clonogenicity and heterogeneity, and (d) the activation of key adhesion proteins, such as integrin. **(B)** Integrin-dependent outside-in signaling mechanisms regulated cell adhesion to ECM as part of their role in cancer radio- and drug-resistance. Many of these mechanisms involve the focal adhesion kinase (FAK). **(C)** The Linker of Nucleoskeleton and Cytoskeleton (LINC) complex is composed of two families: KASH located at the nuclear membrane exterior (NME) and SUN situated in the nuclear membrane interior (NMI). LINC regulates the physical transmission of forces generated by the ECM and cytoskeleton. Moreover, a low expression levels of lamin-A/C is correlated with a high cell migration and an increase of therapy resistance. Cells adjust to mechanical tensions by enhancing the expression level of lamin-A and phosphorylated emerin. LINC complex detaches from the nucleus and cytoskeleton to maintain DNA integrity when cells fail to manage the tension. Created with BioRender.

The ECM of tumors, primarily composed of fibrous tissue, becomes stiffer due to an increase of fiber cross-linking (31, 32). This is in line with the development of desmoplasia during carcinogenesis. Desmoplasia is an intense fibrotic response characterized by the formation of dense ECM (31). Tumors with high desmoplasia are considered to be more aggressive and associated with worse prognosis (31). Reports showed cancer-associated fibroblast to (over-)secrete matrix and modulate tumor phenotype and therapy response (31). These dynamic ECM modifications alter the ECM mechanical properties: degradation, re-polymerization, and alignment, contributing to a re-arrangement of ECM fibers and strain-induced stretching (33–38). In order to remodel the matrix, cancer cells and CAFs release enzymes, such as MMP and lysyl oxidases (LOX), which degrade and crosslink the ECM, respectively (Figure 2A). A structural analysis of the fibrillary collagen revealed the presence of reorganized collagen in the tumor-stromal interphase (39). Moreover, it was demonstrated that an increased collagen alignment and fiber thickness is a negative prognostic marker for cancer, supporting the significance of ECM dynamic in cancer progression (40–43).

ECM stiffness is related to a high malignant tumor phenotype (16). This can be explained by: (a) limited distribution and penetration of drugs (44) and/or (b) alterations in integrin signaling, focal adhesions, Rho/Rho-associated protein kinase (ROCK) pathway activation, as well as actomyosin- and cytoskeletal-dependent cell contractility and increased Ca^2+^ influx through mechanosensitive channels (28, 45, 46). For instance, integrins respond to the force alteration by rearrangement and aggregation in clusters at the plasma membrane. The cluster is composed of multiple mechanosensors (e.g., talin, vinculin), signaling molecules [e.g., focal adhesion kinase (FAK), proto-oncogene tyrosine-protein kinase Src (SRC), Phosphoinositide 3-kinase (PI3K)], adapter proteins [paxillin, LIM, and senescent cell antigen-like-containing domain protein 1 (PINCH1)], and actin linker proteins (e.g., filamin, alpha-actinin), which physically connect integrins to the cytoskeleton. On stiff substrates, the resistance to cellular tension leads to talin stabilization mediated by vinculin binding and also enhances FAK activation. These are some of the key mediators of the transmission of contractile forces to the cytoskeleton (17).

The higher aggressiveness also originates from matrix stiffness-induced epithelial to mesenchymal transition (EMT) being accompanied by cancer cell migration and invasion due to loss of intercellular adhesions (Figure 2A) (47, 48). EMT has been found to be related to treatment resistance (47, 48).

Another feature of the intra-tumoral microenvironment regulated by stiffness is the high interstitial hydrodynamic pressure induced by hypervascularization during tumor development. Such pressures have been found to promote tumor progression by impairing vessel function through constriction, thereby limiting tumor oxygen and nutriment supply, also known as hypoxia (44). Hypoxic tumors are known to be resistant to anticancer therapy, including radiation therapy, chemotherapy, and targeted therapy (44, 49).

Additional determinants of tumor stiffness are genetic mutations as they are not limited to initial driver mutations but encompass a wide genomic variation corresponding to the normal tissue where a tumor arises. High stiffness is correlated with dense collagen matrices resulting in small pore sizes for cells to transverse (50, 51). These events can drive genomic diversity through DNA damaged during migration. Meta-analyses showed that tumors originating from stiff tissues (e.g., lung, skin, bone) have substantially higher somatic mutations and chromosome copy numbers than malignancies originating from soft tissues (e.g., bone marrow, brain) (50). There are three hypotheses stating stiffness to drive genomic instability: (a) stiffness induces cell proliferations, increasing the probability to acquire spontaneous mutations; (b) stiffness increases the frequency of nuclear envelop rupture; (c) invasion of cancer cells through packed-tissue environments causes cell selection with a more aggressive phenotype (50).

Altogether, to gain a better understanding in the dynamics of cancer, it is necessary to uncover the effects cellular and extracellular mechanical properties elicit on tumor growth, metastatic spread and therapy resistance (Table 1). The basis of these events is cell behavior, which profoundly depends on mechanical properties and forces controlling signaling pathways involved in cell differentiation, proliferation, migration, and survival mechanisms (64–69).

**Table 1:** Overview of ECM stiffness-mediated resistance in different tumor entities.

**Tumor entity**	**Method**	**Treatment**	**Stiffness effects on therapy resistance**	**References**
Hepatocellular carcinoma	Polyacrylamide (PA) gels	Cisplatin	**↑**stiffness, ↑resistance	(52)
			↓stiffness, ↑cell dormancy, and stem cells characteristics	
	Alginate gel (ALG) beads	Paclitaxel, 5-FU, and cisplatin	↑stiffness, ↑resistance	(53)
	COL1- coated polyacrylamide gel	Oxaliplatin	↑stiffness, ↑resistance	(54)
	96-well PEG-PC hydrogel platform	Sorafenib	↑stiffness, ↑resistance	(55)
Breast cancer	3D alginate-based hydrogel system	Doxorubicin	↑stiffness, ↑resistance in triple negative cells	(56)
	*In vivo:* heterozygous col1a1^tm1Jae^ (mCol1a1) mice with excessive collagen I accumulation	Rapamycin	↑stiffness, ↑resistance in lung metastatic cells but not in primary tumor	(57)
Melanoma	PEG hydrogels	PLX4032	Cell line-dependent response, ↓stiffness, ↑apoptosis	(58)
	*In vivo* serial biopsies	Vemurafenib (Zelboraf®)	↑stiffness, ↑resistance, ↑tumor relapse	(59)
Myeloid leukemias	3D hydrogels, *in vivo*	Several chemotherapeutics	↓stiffness, ↑resistance to standard chemotherapeutics	(60)
Laryngeal squamous cancer	Polyacrylamide (PA) gels	Cisplatin or 5-FU	↓stiffness, ↑resistance	(61)
Pancreatic cancer	Polyacrylamide (PA) gels	Paclitaxel	↑stiffness, ↑resistance	(62)
Glioblastoma	Chitosan–hyaluronic acid scaffolds	Temozolomide	↑stiffness, ↑resistance	(63)

*Research methods and treatment are included*.

The role of the ECM in treatment resistance was predominantly investigated for chemotherapeutics, with breast cancer being one of the most frequent models used (Table 1). As a matter of fact, one of the early detection methods for this tumor entity was the determination of abnormal stiffness by palpation or using medical devices. By means of an poly(ethylene glycol)-phosphorylcholine (PEG-PC) hydrogel system, Nguyen et al. examined the response of breast cancer cells to the Raf kinase inhibitor sorafenib in different stiffness substrates (55). The efficacy of sorafenib was reduced depending on stiffness and collagen concentration but independent of the commonly associated ROCK activity. Instead, triple negative breast cancer cells sustained an activation of JNK mediating the drug resistance (55). However, the combination of a JNK inhibitor with sorafenib eliminated the stiffness-mediated resistance. Strikingly, they found out that ERK (extracellular-signal-regulated kinase) and p38 (mitogen-activated protein kinases) were not involved in the drug resistance, it was rather regulated by β1 integrin (55).

Moreover, Joyce et al. showed that extrinsic resistance is associated with matrix stiffness (56). As culture model, an innovative 3D alginate-based hydrogel system enabling dynamic ECM stiffening over time was used. The results displayed a stiffness-dependent response to the chemotherapeutic doxorubicin in triple negative breast cancer cells (MDA-MB-231), while a non-triple negative cell model (MCF7) failed to show the same stiffness-dependent resistance (56). This differential therapeutic response was correlated with a nuclear translocation of YAP, a marker of mesenchymal differentiation. In fact, a higher level of nuclear YAP was found in MDA-MB-231 relative to MCF7 cells (56).

Another example of a cancer entity with poor prognosis that seems to be dependent on ECM stiffness and EMT is pancreatic cancer (62). Pancreatic cancer is one of the stiffest human solid carcinomas characterized by a fibrotic reaction, leading to the activation of EMT-related and prosurvival signaling pathways (62). Rice et al. reported that *in vitro* PDAC cell lines cultured on varying stiff polyacrylamide gels had different behavior than the corresponding tumors *in vivo*. Resistance to gemcitabine, a therapeutic drug that inhibits DNA synthesis and transcription, was shown to be unchanged with increased rigidity, although matrix rigidity still promoted EMT. In contrast, cells grown on stiff gels showed increased resistance to paclitaxel (a taxane that stabilizes microtubules preventing mitosis) compared with the softer conditions (62).

The second most studied tumor entity, in terms of matrix stiffness, is the hepatocellular carcinoma (HCC) since it often relates to liver fibrosis. Various studies demonstrated resistance to cisplatin, sorafenib, paclitaxel, 5-FU, and oxaliplatin to depend on ECM stiffness (52–55). It was also shown that a large number of cells were dormant and carrying stem cell-like characteristics in HCC when cultivated in low stiffness (52). Liu et al. cultured HCC cells in alginate gel beads with different degrees of stiffness (53). Cells cultured in the stiff matrices resisted to cisplatin, 5-FU, and paclitaxel, whereas cells in the soft environment were sensitive to these agents. Moreover, cells encapsulated in alginate beads highly express ABC transporters and endoplasmatic reticulum-related proteins compared to 2D growth conditions. These proteins are supposed to contribute to drug resistance of solid tumors and treatment failure.

A recent study focused on the matrix stiffness-mediated effects in HCC stem cells (54). In this work, the authors showed that, when the substrate stiffness is increased, HCC cells exhibit an elevated number of CD133(+)/EpCAM(+) positive cells (stem cells markers). The increase in this cell population was accompanied by elevated expression levels of EpCAM, Nanog, and SOX2 (54). Moreover, the phosphorylation levels of Akt and mTOR were upregulated showing a greater self-renewing ability and oxaliplatin resistance. Interestingly, when these populations were subjected to integrin inhibition, all the previous described effects were attenuated, suggesting that integrin β1 may deliver higher stiffness signal inside HCC cells activating stemness associated signaling cascades (54).

Opposite to the results from You et al. (54), human laryngeal squamous cell carcinoma (Hep-2) cells cultured in a low stiffness environment showed an enhanced expression of stem cell markers (61). In addition, under the low stiffness environment, Hep-2 cells underwent less apoptosis to cisplatin and 5-FU. The authors suggested that the observed chemoresistance is related to increased Sox2 levels and an upregulation of the ABCG2 protein, a membrane xenobiotic transporter connected to multi-drug resistance (61). These examples illustrate the diversity of resistance mechanisms in different tumor entities, suggesting that there is no “one-for-all” approach, and thus only tumor-specific studies shed light on the mechanisms.

Tokuda et al. studied the effect of stiffness on the treatment response of melanoma cells, showing a cell-line dependent effect (58). Cells were grown in different PEG hydrogels with variable tensile moduli and treated with a BRaf inhibitor—PLX4032. Cells derived from radial growth phase (WM35) presented stiffness-dependent chemoresistance in contrast to the metastatic melanoma cells (A375) (58). A recent study on therapeutic relapse to another BRaf inhibitor—vemurafenib—used serial biopsies of genetically modified mice (59). Next-generation sequencing and single-cell transcriptomics enabled tracking of the evolution of multiple cellular “compartments” within individual lesions during first-line treatment response, relapse, and second-line therapeutic interventions (59). It became clear that tumor relapse is genetically stable, while differentiation state and ECM contribute significantly to the resistant phenotype. The result from *in vitro* experiments presented a correlation between cell state changes and ECM remodeling, suggesting an increased tumor stiffness modulates tumor cell fate and reduces treatment responses (59).

For glioblastoma, the most common brain tumor in adults (70), no physiologically relevant model is currently available for exploring effects of cellular stiffness. The majority of investigations on stiffness applied 2D cultures system. Erickson et al. suggested a newly developed and characterized Chitosan-Hyaluronic Acid scaffold with varying stiffness for glioblastoma cell culture (63). They showed glioblastoma cells to form large spheroids in stiff scaffolds exhibiting a higher degree of drug resistance and a more invasive phenotype relative to 2D models (63).

Altogether, we conclude that an increase of ECM stiffness leads to enhanced therapy resistance, with some exceptions that might be tumor- or substrate/matrix-dependent. ECM stiffness, therefore, might be used as a physical marker for the prediction of tumor therapy resistance. Certain contradictory issues, especially in terms of stemness, need to be clarified. Cancer stem cells are a well-known factor of therapy resistance and more studies are necessary to understand how these subpopulations behave in different stiffness substrates.

## Regulation of Cancer Resistance Through Cellular Stiffness

Regulation of cellular stiffness is typically dictated by a variety of factors such as cytoskeleton organization, number of focal adhesion clusters, and nuclear deformability. Generally, cancer cells tend to be softer than their normal counterpart (= tissue of origin) depending on the status of their malignant transformation (35, 71–77).

Using magnetic tweezers to probe cellular resistance to physical force, a study in ovarian cancer cells demonstrated that the migration and invasion potential are inversely proportional to cellular stiffness. Moreover, some treatments such as pharmacological myosin II inhibitors reduce cellular stiffness and, therefore, convert cancer cells into a more invasive phenotype (75, 78). Pathways regulating these mechanical cues may potentially serve as targets for molecular cancer therapy.

Cellular stiffness is also determined by particular membrane proteins found in focal adhesions. FAPs assemble into protein complexes and act as connecting and adaptor proteins between ECM and the cellular interior (18–20). The complexes transmit extracellular signaling and mediate a strong interaction with the actin cytoskeleton. In many cancers, these proteins are de-regulated, resulting in abnormal cell-cell and cell-ECM adhesion. Integrins are commonly overexpressed in tumors and affect growth rate, cellular morphology, and invasiveness (28, 79, 80). Integrin activation triggers cytoskeletal re-arrangements through the regulation of signaling cascades like Src- and FAK and their downstream signaling pathways for therapy resistance (81).

The effects of cellular biophysical properties fundamental for therapy resistance remain to be clarified (Table 2). Liu et al. used a microfluidic platform to evaluate cancer cell transportability and invasiveness in heterogeneous breast cancer cells (90). Cell transportability is determined by cellular stiffness and cell surface frictional property, allowing the discrimination between more and less invasive phenotypes (90). The same principle was applied in another study. Leukemic cells treated with daunorubicin were sorted according to their cellular stiffness using a microfluidic device (88) uncovering cellular physics to serve as distinctive features between chemoresistant and -sensitive cells. Softer cells showed an alteration in multiple mechanisms related to drug resistance, including decreased sensitivity to apoptosis induction, enhanced metabolic activity, and regulation of key genes involved in extrusion of drugs such as CYP supergene family typically involved in drug resistance (88).

**Table 2:** Cell stiffness and related causes in different tumor entities, together with an overview of the methods for measuring cell stiffness.

**Tumor entity**	**Method**	**Treatment**	**Stiffness effects on therapy resistance**	**References**
Breast cancer	Optical tweezers	Different stiffness substrates	↓ECM stiffness, ↑cellular stiffness	(82)
	Atomic Force Microscopy (AFM)	Soft and stiff gels//EGFR inhibitor (Cetuximab)	↑stiffness when cultured on stiffer substrates//↑stiffness upon EGFR inhibition	(83)
Prostate cancer	Magnetic twisting cytometry	Paclitaxel	↑stiffness, ↑resistance, ↑fluid-like behavior	(84)
Ovarian cancer	Atomic Force Microscopy (AFM)	Cisplatin	↑stiffness, ↑resistance	(85)
	Filtration device depending on cellular pressure driven deformation	Cisplatin	↓stiffness, ↑resistance	(86)
Liver cancer	Atomic Force Microscopy (AFM)	Different shear stresses (parallel plated flow chamber)	↑shear stress, ↓stiffness	(87)
Leukemia	Microfluidic system for cell sorting and Atomic Force Microscopy (AFM)	Daunorubicin	↓stiffness, ↑resistance	(88)
Transformed mesenchymal stem cells	Atomic Force Microscopy (AFM)	Hypermethylation of cancer 1 (HIC1) and Ras-association domain family member 1A (RassF1A)	↓stiffness, ↑tumor aggressiveness	(89)

Using lab on chip technology, several studies investigated the influence of cell deformability on the chemotherapeutical response of ovarian, breast, and prostate cancer. It became clear that cisplatin-resistant ovarian cancer cells have more elastic deformation capability relative to cisplatin-sensitive cell populations (86). Although these results seem exciting, they are in contrast to the study of Sharma et al. showing that cisplatin-resistant ovarian cancer cells are stiffer than their normal counterpart. This stiff phenotype is characterized by cytoskeletal long actin stress fibers mediated by Rho GTPases (85).

In line with this, paclitaxel-resistant prostate cancer cells were shown to be stiffer than the non-resistant counterpart. Kim et al. showed that paclitaxel-resistant cells gain mobility and invasiveness through increased EMT (84). Moreover, enhanced cell migration and invasion of paclitaxel-resistant cells was facilitated by increased cytoskeleton remodeling dynamics, stiffness, traction forces, and by a repression of keratin 8/18/19. In this work, the authors observed that resistant prostate cancer cells, despite being stiffer than the non-resistant cells, showed a more fluid-like behavior leading to a higher invasion capability (84).

In another study, matrices of different stiffness were used to understand the cellular behavior of different breast cancer cell lines (82). Interestingly, the most aggressive cells (MDA-MB-231) were softer when cultured on glass substrate, but when these cells were cultured on soft matrices they presented a stiffer phenotype compared to the other cell lines cultured in the same matrix. This is a good example of how the environment modulates cellular mechanical properties (82).

A similar work on breast cancer cells using matrices of different rigidity discovered a direct correlation between migration capacity and increase of matrix stiffness (83). Moreover, cells treated with cetuximab, an epidermal growth factor receptor (EGFR) inhibitor, had an increased elastic modulus followed by a decrease in migration ability. Here, the authors explained that cell mechanics are not only regulated by mechanical cues of the ECM but also by biochemical signals mediated through membrane receptors, such as EGFR (83).

Another study investigating environmental effects on liver cancer stem cells is from Sun et al. The authors investigated the effects of shear stress on cancer stem cell signaling regulating cellular migration, proliferation, and differentiation (87). It was found that certain shear stresses promote cell migration through activation of FAK and ERK1/2 signaling pathways. Moreover, shear stresses were responsible for lowering cellular stiffness in line with disrupted F-actin organization (87).

Environmental factors can also regulate epigenetic signatures such as methylation (89). Using cell lines with methylated tumor suppressor genes (e.g., hypermethylated in cancer 1—HIC1, Ras-association domain family member 1A—RassF1A), a Taiwanese group investigated cell stiffness changes depending on the methylation status and found that the stiffness of the methylated cells was lost, followed by a decrease of tubulin expression and F-actin disorganization (89). Further experiments involving cellular relaxation after cell compression showed that cancerous cells also have increased acto-myosin cortex contractility as compared to corresponding healthy cells (74, 91). Moreover, the higher the invasive level, the greater the cellular recovery behavior.

In contrast to ECM stiffness, cellular stiffness seems not to correlate with treatment resistance. Although there is a prevalence that a decrease cellular stiffness leads to an increase resistance, this assumption is often uncertain due to several factors: (1) measurement technique, (2) cell culture methodology, and (3) tumor entity/heterogeneity.

## Integrins Bridging Between ECM and Cellular Stiffness: Effects on the Resistome

After years of research, it became obvious that cell adhesion is fundamental for cell survival (92). Furthermore, a number of studies showed that cell adhesion is associated with the refractory to cancer treatments (92, 93). The principles of treatment resistance of cancer modulated by cell adhesion were proposedly categorized into: (i) cell adhesion mediated radioresistance (CAM-RR) and (ii) cell adhesion mediated drug resistance (CAM-DR) (Figure 1). Diverse adhesion resistomes composed of integrins, adaptor proteins, kinases, and cytoskeleton mainly contribute to both resistance mechanisms (92, 93). Interestingly, the components of the adhesion resistomes are widely heterogeneous depending on tumor entities. These might be also related to the tissue of origin.

To form multicellular structures or tissues, cells need to attach to adjacent cells via cell-cell contacts and anchor to the ECM through the transmembrane adhesion receptors known as integrins. An integrin receptor is a non-covalent heterodimer consisting of an α and a β integrin subunit. To date, there are 18 α and 8 β integrin subunits allowing the formation of 24 different integrin receptors. These α and β combinations determine the binding specificity of the integrin (29). Essentially, integrins consist of a big extracellular ectodomain, a transmembrane domain and a short cytoplasmic tail (29).

In the last two decades, substantial studies on cell adhesion to ECM primarily focused on integrins. Integrins and their downstream FAPs are known as mechano-sensors and mechano-transducers that sense and transduce mechanical signals into chemical signals. Generally, normal tissues weakly express integrins and FAPs. In contrast, cells start to express them when cells are grown in an *in vitro* tissue culture surfaces, indicating that cell culture stiffness highly impacts on the expression of these proteins (94).

Most integrins are not constitutively active and are located at the cell surface in an inactive state. Integrins are bi-directional signal receptors stimulated in two ways: the inside-out and outside-in activation (95). Both activation pathways are based on a conformational change in the ectodomain of the integrin (Figure 2B). The ability of integrins to signal in an inside-out and outside-in manner may be exquisite in normal cells but it is deleterious in cancer cells (92). The outside-in signaling is better understood with regard to its role in the cell adhesion resistome to elicit CAM-DR and CAM-RR compared to the inside-out signaling, which is rarely investigated in cancer cells (96–107).

During the inside-out signaling, the cytoplasmic domain of the integrin binds and stimulates intracellular proteins such as kindlin or talin. By integrin conformational changes, there is an increased binding affinity for extracellular ligands. This activation mechanism controls, among other things, the migration of cells. With the help of outside-in activation, which is mainly dictated by ECM properties, integrins can introduce information into the cell. The extracellular binding of a ligand also leads to conformational changes of the integrin and activation of intracellular signaling pathways (Figure 2B). Often this signaling pathway recruits and activates kinases such as FAK and SCR, and also the RAS-MAPK (mitogen-activated protein kinase) and PI3K (phosphoinositide 3-kinase)—AKT (RAC-alpha serine/threonine Protein kinase) signaling nodes (42). Moreover, both signaling pathways, inside-out and outside-in, are powerful and can activate each other (108–114).

Binding of integrins to ECM proteins is mediated by short amino acid sequences. Motifs that can bind these sequences are (1) the RGD (arginine-glycine-aspartate) motif in fibronectin and laminin or (2) the DGEA (aspartate-glycine-glutamic acid-alanine)—and the GFOGER (glycine-phenylalanine-glycine-glutamic acid-arginine) motif in collagen (29, 115). Intracellular adapter proteins such as paxillin, parvin, or talin link integrins to the actin cytoskeleton, generating a bridge between ECM and cytoskeleton. Although integrins do not have intrinsic kinase activity, they recruit and activate a large spectrum of kinases to the cytoplasmic subunit. As a result, important cellular processes such as proliferation, apoptosis, differentiation, migration, or cell survival regulated (Figures 1, 2B) (92, 116–119).

Beta-1 integrins are the largest subgroup of integrin adhesion receptors (29). Inhibition of β1 integrins, leads to an inactivation of a variety of integrin receptors such as for laminins, fibronectin, and collagens. This property, as well as the upregulated expression of β1 integrins in a variety of tumors, make β1 integrins a promising target molecule for cancer therapy (92, 120). The resistance of tumors to radiation and chemotherapy is dependent on the β1 integrin adhesion to ECM proteins. A collection of studies showed the importance of β1 integrin-mediated pathways for radiation resistance and survival, differentiation and proliferation, as well as for tumor progression and metastasis (34, 100, 101, 106, 107, 121–126).

In clinical trials, some inhibitors against β1 integrin receptors have been used. These include three inhibitors against the fibronectin receptor α5β1 integrin: ATN-161, Volociximab (M200) and JSM6427. ATN-161 is a peptide, which acts as an antagonist of the α5β1 integrin and blocks the receptor. Phase I studies showed that the use of ATN-161 had no risks or side effects (127). Volociximab, a humanized monoclonal antibody, has been reported as an angiogenesis inhibitor developed for solid tumors. Treatment with volociximab was in Phase I and no adverse reactions nor dose-related toxicity was observed (127). Further clinical studies in metastatic melanoma and renal cell carcinoma have shown promising effects upon volociximab treatment (128). The third α5β1 integrin inhibitor is the small molecule JSM6427 which inhibits angiogenesis and fibrosis and has so far only been tested in preclinical studies (127, 129).

Therefore, preclinical examinations have already described the importance of β1 integrin-mediated adhesion to ECM for survival of tumor cells after irradiation. Studies in several tumor entities were able to demonstrate that the inhibition of β1 integrin leads to radiation sensitization in glioblastoma cells (81, 130), lung carcinoma cells (131), colon carcinoma cells (132), breast carcinoma cells (133, 134), and HNSCC cells (121, 135). *In vitro* data from 3D cultured cells and data from xenograft tumors confirmed that inhibition of β1 integrins reduces significantly the radiation resistance of tumors (121, 134).

Depending on the integrin receptor and the tumor entity, integrins activate different survival-promoting signaling pathways. In breast cancer cells, the PI3K-AKT signaling pathway is mainly activated leading to adhesion-mediated radiation resistance (134). Integrins modulate the FADD (caspase-8/Fas-associated protein with death domain) signaling pathway, which is of importance for cell survival, resulting in the resistance to radiation induced cell death in leukemia cells (136). In HNSCC, FAK is the central signaling molecule for the β1 integrin-mediated signaling pathways and plays an essential role for cell survival after irradiation (116, 121). Data from our group showed that the inhibition of β1 integrin dephosphorylates FAK, causing the FAK/cortactin complex dissociation. This leads to the inactivation of JNK1 and the radio-sensitization of tumor cells (121). FAK consists of an N-terminal FERM (protein 4.1, ezrin, radixin, moesin homology) domain, a kinase domain, and a FAT (focal-adhesion targeting) domain (137, 138). The FERM domain mediates various interactions of FAK, e.g., with the EGFR. The FAT domain is responsible for the recruitment of FAK to the focal adhesion site. It binds integrin-associated adapter proteins such as talin or paxillin. In addition, FAK contains three proline-rich ones Regions (PRR1-3) that help proteins target a SH3 (SRC-homology 3) domain contained as e.g., p130Cas (139).

FAK can be phosphorylated on various tyrosine residues (139). The autophosphorylation of the tyrosine 397 site is triggered through the bond of β1 integrin to the ECM. In HNSCC cells, inhibition of β1 integrins leads to the dephosphorylation of FAK on tyrosine 397. This phosphorylation site is therefore used as a control of a functional β1 integrin inhibition (121). Interestingly, Lim and colleagues identified that FAK plays an important role in the nucleus. They showed that p53 binds to the FERM domain of FAK, thereby modulating cell survival and proliferation (140, 141). This observed function in the nucleus suggested that FAK has additional nuclear functions and, thus, might contribute to the rectification of radiation-induced DSB. In line, our group demonstrated that the non-homologous end-joining DNA double strand break repair pathway is partially co-controlled by β1 integrins via the FAK/JNK1 signaling axis (100). The significance of integrins in DNA repair processes was further emphasized by Christmann and colleagues. They observed that the αV/β3 integrin signaling axis coordinates the homologous recombination repair pathway in glioblastomas (142). Upon simultaneous temozolomide treatment and αV/β3 integrin knockdown, glioblastoma cells presented increased DNA double strand breaks and a depletion of Rad51 expression, indicating an impaired homologous recombination (142).

Furthermore, we have shown that β1 integrin targeting leads to an induction of the EGFR signaling cascade and the double targeting of β1 integrin and EGFR achieved a greater radiosensitization compared to the single targeting approaches *in vitro* and *in vivo* (101). This suggests a more efficient suppression of FAK/ERK (extracellular-signal-regulated kinase) prosurvival signaling upon the combination treatment of anti-β1 integrin/anti-EGFR treatment than the single therapy.

To date, only a few studies attempted to investigate the crosstalk between integrins and receptor tyrosine kinases (RTK) and its effect on cancer cell therapy resistance. Therefore, more studies are needed to identify the therapeutic potential of such combination therapy approaches. One of our recent study showed that HNSCC cells, which basically poorly respond to EGFR and β1 integrin blockage, were radiosensitized by the inhibition of targets identified from a whole exome sequencing (123). Briefly, we identified different gene mutation profiles in the non-responder HNSCC cell lines to EGFR and β1 integrin inhibition compared to the responder HNSCC cell lines. These profiles would allow the stratification of HNSCC patients and the identification of potential targets to address the treatment resistance. Kelch Like ECH Associated Protein 1 (KEAP1) and Mammalian target of rapamycin (mTOR) were identified as key targets. The pharmacological inhibition of KEAP1 or mTOR together with β1 integrin and EGFR effectively increased non-responder radiosensitization (123). The study suggested a therapeutic approach to identify a potential combination therapy and to promote identifications of novel targets.

In summary, we can assert that integrins and FAPs essentially contribute to therapy resistance and the possibility of targeting these proteins could be developed as a therapeutical option in combination with radiotherapy and chemotherapy.

## Regulation of Cancer Resistance Through Nuclear Stiffness

During tumorigenesis, in addition to altered ECM stiffness, contractility of the cytoskeleton, and cell adhesion, stiffness of the cell's nucleus actively or passively adjusts to the process of malignant transformation. A growing number of studies report a modified nuclear envelope structure and composition in cancer cells (143). The nuclear envelope, consisting mainly of lamins and nuclear pore complexes, was identified as the major structure that is modulated in cancer (143, 144). The nuclear envelope contributes to cellular mechanical properties and functions and determines nuclear deformability (145). It is also involved in mechano-transduction and transmission of forces to the nucleus. Cancer progression promotes modifications in the composition of the nuclear envelope generating softer and highly lobulated nuclei, which consequently allow cancer cells to invade dense tissues more easily (143, 146). The nuclear stiffness is predominantly modulated by mechano-signals communicating between ECM and nucleus. Physical interactions of nucleus and cytoskeleton are essential for cytoskeletal organization and cellular polarization, which influence cell migration for metastasis (147). Moreover, the interaction seems to induce rearrangements in chromatin structure and lamin expression via intranuclear signaling cascades (143, 144, 146).

A study using a microfluidic channel with a narrow constriction to investigate the stiffness of prostate cancer nuclei showed that the nuclear rigidity is reduced in more malignant phenotypes. Furthermore, prostate cancer cells expressed a more aggressive phenotype when a low expression level of lamin-A/C and a decreased chromatin condensation were present (148). This supports the hypothesis that cancer cells with softer nuclei metastasize more efficiently. The importance of nuclear stiffness in cellular migration was also shown in lung carcinoma and glioblastoma multiforme. Generally, lamin-Bs are more stably expressed than lamin-A, of which the expression level widely varies among normal and cancerous solid tissue cells. In this study, cells with low levels of lamin-A expression showed the most pronounced increase in 3D migration. Of key importance was the finding that the cellular migration was biphasic in lamin-A expressing cells as wildtype lamin-A protects cells against stress-induced cell death. In fact, knockout of lamin-A caused broad defects in stress resistance. Therefore, lamins impede 3D migration but also promote survival against migration-induced stress (149).

Remodeling of the nuclear structures is associated with mechanical stress transmitted via the ECM/FAPs/cytoskeleton/nuclear envelop protein axis. The mechanical stress transmission axis promotes the epigenetic changes and the modification of chromatin dynamics that influence on the nuclear behavior (150). FAPs, however, can become activated independent of ECM in certain cases e.g., in breast cancer cells (151, 152). During tumor progression, microenvironmental control of nuclear organization seems to be impaired but still dependent on β1 integrin signaling (152).

Of great interest is a finding showing that DNA repair proteins are mechanosensitive factors leading to a new field of mechano-genomics (153). The group of Discher focuses on the spatiotemporal changes of endogenous DNA damage and repair factors in cells migrating through rigid micropores and on the lasting perturbations to the genome. The study showed that multiple DNA repair proteins avoid mechanical stress upon pore migration, resulting in a cytoplasmic mislocalization sustained for many hours, which leads to delayed repair and consequently DNA sequence alteration (154, 155).

In the previous section, we discussed about signaling cascades activated from the integrin axis. These mechanical signals are then transduced to the nucleus though mechanosignaling, in other words biochemical mechanotransduction pathways. In addition to the mechanosignaling, there is a faster way to transmit physical signals directly to the nucleus possibly through the physical anchoring of the cytoskeleton with the nuclear lamina via the linker of nucleoskeleton and cytoskeleton (LINC) complex (17). This complex is composed of two family members, which are SUN (Sad1p, UNC-84) and KASH (Klarsicht/ANC-1/Syne Homology) located at the interior and the exterior of the nuclear membrane, respectively (Figure 2C). Typically, SUN is connected with the nuclear intermediate filament lamins, whereas, KASH interacts with cytoskeletal proteins, such as intermediate filaments, actin filaments and microtubules. SUN and KASH proteins interact within the perinuclear space forming a bridge between cytoskeleton and nucleoskeleton (156). Guilluy and colleagues studied the association of LINC complex with mechanical tension. They showed that an isolated nucleus adapts to mechanical tension induced by magnetic tweezers, which results in increased nuclear stiffness. The stiffening of the nucleus did not involve structural modification of chromatin or nuclear actin, but required an intact nuclear lamina and phosphorylation of emerin, a protein of the inner nuclear membrane (157).

In a recent study from the Swift group, the response of cells to cyclic tensile strain mimicking the dynamics of the microenvironment *in vivo* was investigated (158). A series of strains with different intensities was applied to cells. They observed that cells subjected to low levels of strain responded similar to cells exposed to an increased stiffness. In case cells were exposed to the high intensities, the composition of LINC complex was altered, specifically the SUN domain containing the SUN2 protein. This domain was significantly affected by protein levels and posttranslational modifications leading to a strain induced breakpoint in the linker complex. As a result, cells were able to detach the nucleus from the cytoskeleton in case of excess stress, conferring a protection to DNA (158).

Collectively, nuclear stiffness is associated with tumor aggressiveness, especially in migration and metastasis. However, more studies are required to understand the underlying mechanisms and to validate whether nuclear stiffness can be used as a predictive biomarker of therapy response.

## Concluding Remarks and Perspective

We discussed recent studies showing how the tumor creates a microenvironment favorable for proliferation, invasion and treatment resistance. Cellular, nuclear, and ECM stiffness play essential and intertwined roles in the cancer response to therapy. Despite many investigations performed with regard to the impact of stiffness on chemotherapy response, it remains open if these results are similar and can be translated to the response to radiotherapy. We have shown that the presence of a 3D environment and matrix composition affects radiotherapy response upon the activation of FAPs (CAM-RR) (Figure 1) for pro-survival signaling. FAPs and extracellular matrices have been defined as important determinants of the hallmarks of cancer (30, 101, 159–161). In our previous studies, we demonstrated different growth conditions to modulate chromatin structure, DNA repair and cell survival upon radiation exposure (100, 162). Obviously, force transmission and mechanotransduction are mediated by FAPs to enable control over nuclear processes including therapy resistance. Together, the current body of literature strongly supports the concept of mechanical characteristics of the cellular environment to critically regulate the epigenetic and genetic landscape driving cancer cell radiochemoresistance.

Clearly, the matrix stiffness is a main element in cancer therapy resistance, especially in chemotherapy. Radiotherapy typically induces fibrotic reactions that, consequently, amplify tissue stiffening. This causes complications in normal tissues such as lung fibrosis. A combination of multiple factors like fibroblast activation, vascular damage, and leakage, etc., promotes ECM remodeling and excess matrix deposition (163–165). To date, it remains to be understood to what extent these therapy-induced changes contribute to tumor progression, resistance, and metastasis.

The role of stiffness in resistance and the potential of ECM, cellular, and nuclear stiffness as a biomarker for therapy response are still elusive. This ambiguity is also due to the heterogeneous set of data, which may sometimes be conflicting (Table 2 provides some examples). Therefore, an optimized and standardized approach for the study of stiffness is necessary. Moreover, it would be of great benefit for the community to collaboratively standardize experimental setups and measurement techniques.

Despite the number of research groups studying cell behavior on different substrates with different stiffness, the impact of these matrices on cell function and therapy response has only recently been appreciated. Future efforts may focus on (1) how stiffness sensing occurs in different macro-micro-nano-scales (ECM/tissue, cell, nucleus) and (2) whether stiffness is a promising biomarker for therapy response or even a therapeutic target.

## Author Contributions

SD and NC formulated the topic of the review and drafted and approved the manuscript.

### Conflict of Interest

The authors declare that the research was conducted in the absence of any commercial or financial relationships that could be construed as a potential conflict of interest.
